# Foveal vision reduces neural resources in agent-based game learning

**DOI:** 10.3389/fnins.2025.1547264

**Published:** 2025-03-11

**Authors:** Runping Chen, Gerd J. Kunde, Louis Tao, Andrew T. Sornborger

**Affiliations:** ^1^Center for Quantitative Biology, Academy for Advanced Interdisciplinary Studies, Peking University, Beijing, China; ^2^Los Alamos National Laboratory, Los Alamos, NM, United States; ^3^Center for Bioinformatics, National Laboratory of Protein Engineering and Plant Genetic Engineering, School of Life Sciences, Peking University, Beijing, China

**Keywords:** neuromorphic computing, visual neuroscience, multi-resolution sensory integration, neural resources, reinforcement learning

## Abstract

Efficient processing of information is crucial for the optimization of neural resources in both biological and artificial visual systems. In this paper, we study the efficiency that may be obtained via the use of a fovea. Using biologically-motivated agents, we study visual information processing, learning, and decision making in a controlled artificial environment, namely the Atari Pong video game. We compare the resources necessary to play Pong between agents with and without a fovea. Our study shows that a fovea can significantly reduce the neural resources, in the form of number of neurons, number of synapses, and number of computations, while at the same time maintaining performance at playing Pong. To our knowledge, this is the first study in which an agent must simultaneously optimize its visual system, along with its decision making and action generation capabilities. That is, the visual system is integral to a complete agent.

## 1 Introduction

The sensorimotor system is the essential pathway through which organisms sense and interact with their environment via rapid and efficient adaptive responses to incoming sensory information. Understanding the neuronal and neural circuit mechanisms by which organisms develop and implement effective strategies to navigate external challenges is a pivotal topic in the biological sciences. This process, often driven by plasticity and learning, involves a continual adjustment of behavior based on experience. Reinforcement learning (RL), which trains behavior through rewards, has a well-established biological foundation and is integral to adaptive learning and decision-making (Montague et al., 1992, 1996; Schultz et al., 1997; Day et al., 2007; Knutson et al., 2009; Niv, 2009; Lee et al., 2012; Kable and Glimcher, 2009).

The principles underlying reinforcement learning are not only fundamental to biological systems but are also highly relevant for developing advanced artificial intelligence (AI) and robotics. In AI, RL algorithms have demonstrated remarkable capabilities, including equaling or surpassing human performance in various complex video games (Mnih, 2013; Wurman et al., 2022; Baker et al., 2022; Vinyals et al., 2019), robot manipulation (Nguyen and La, 2019), and chip design (Mirhoseini et al., 2021). These advances suggest that artificial neural networks (ANNs) can be designed to reproduce certain aspects of biological learning, potentially leading to adaptive and intelligent AI systems. For robotics, understanding and implementing these principles could enhance the ability of robots to learn from their environment and improve their interaction capabilities in complex, dynamic and unpredictable settings. The difficulty of balancing rich visual input with efficient resource usage has been studied previously in RL agents (for example, Mnih, 2013 scaled down visual input from 210 × 160 × 3 to 80 × 80 to reduce computations).

In biological organisms, sensory input plays a critical role in shaping the downstream neural network. Sensory experiences influence the development and organization of neural circuits, driving the refinement of sensory processing and motor responses. This input not only impacts the initial formation of synaptic connections but also continuously modulates neural plasticity, allowing the brain to adapt to new information and changing environments (Wiesel and Hubel, 1963; Holtmaat and Svoboda, 2009; Hübener and Bonhoeffer, 2014). The ability of neural networks to reconfigure based on sensory feedback is essential for learning and adapting behaviors, highlighting the intricate relationship between sensory experiences and neural development.

The visual system, one of the major sensory systems for mammals, receives and processes optical information with a spatially-variant structure (Land and Eckert, 1985; Bringmann et al., 2018; Bringmann, 2019), combining high-resolution foveal vision and low-resolution peripheral vision. This design, alongside eye movements, allows organisms to efficiently gather environmental information.

Researchers have explored ways to replicate biological multi-resolution visual systems to enhance computational efficiency. In terms of sampling methods, some models adopt a geometric approach (Traver and Bernardino, 2010; Killick et al., 2023; Liu et al., 2024), reducing sampling rates from the vision center outward. For example, Lukanov et al. (2021) use Foveal Cartesian Geometry (FCG), in which pixels are sampled from concentric squares around the fovea, to emulate retinal sampling. Then FCG foveated images are used as the input a CNN classifier. Other researchers use a simple two-resolution strategy (Chipka et al., 2021; Min et al., 2022): a uniform low-resolution image for peripheral vision and a small high-resolution image cropped for foveal vision. The model of (Xia et al., 2020) uses low-resolution video frames as the peripheral visual input to predict human driver gaze and uses high-resolution image patches from the predicted gaze locations. Both peripheral and foveal input are used to predict the vehicle speed at high accuracy and high efficiency.

Simulating eye movements is a key focus to build multi-resolution visual systems. Xia et al. (2020), Zhang et al. (2020), and Rong et al. (2021) studied imitation learning. Thakur et al. (2021) proposed Gaze Regularized Imitation Learning (GRIL), which learned concurrently from both human demonstrations and eye gaze to solve a visual navigation task. They showed that GRIL outperformed several state-of-the-art imitation learning algorithms. A second approach relied on attention maps from convolutional neural networks (CNNs) (Killick et al., 2023). Lukanov et al. (2021) to extract an attention map from the final convolutional layer and used it for bottom-up attention utilizing salient features from the observations. The classification output assisted a top-down attention mechanism to augment the attention map. The maximum intensity in the attention map was used to locate the foveal position. Another approach employed RL to dynamically control eye movements based on task demands (Liu et al., 2024). To capture more objects in human-driving video, Chipka et al. (2021) trained a Deep-Q Network, where true positive detections gave a reward. This foveal attention method provided a considerable improvement in performance for vehicle detection.

**Figure 1:**
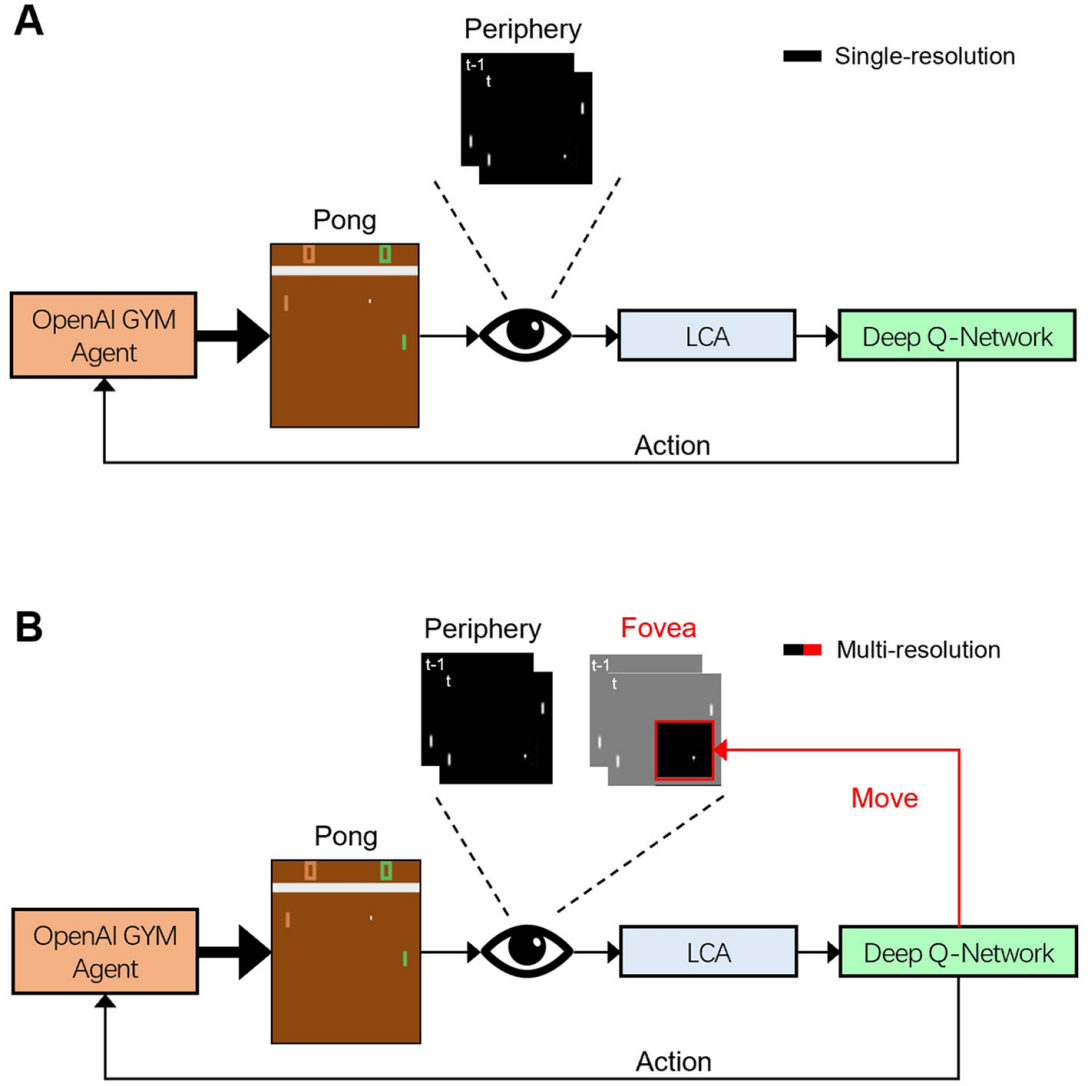
Schematic diagram of the single- and multi-resolution Deep-Q Network (DQN) agents that learn to play Pong. Open AI Gym Pong video frames were preprocessed into a peripheral, low-resolution but full frame and a fovea-like, high-resolution but zoomed frame (active areas are denoted in black). These frames from up to four time steps (labeled by “*t, t* − 1, ...") were sparse coded by a Locally Competitive Algorithm (LCA). The DQN agent derived actions from these outputs. The single-resolution agent **(A)** exclusively received visual input from the periphery, then determined the paddle action, which was sent back to the OpenAI Gym agent (black “Action” arrow). The multi-resolution agent **(B)** received input from the periphery and fovea and returned the paddle action to OpenAI Gym and additionally updated the foveal movement parameters, used internally in the agent for the next time step (black “Action” and red “Move” arrow, respectively).

Here, we propose a novel framework (Figure 1) for studying sensorimotor systems using reinforcement learning. We introduce a multi-resolution visual system with a Locally Competitive Algorithm (LCA) (Rozell et al., 2008) front end, that closely emulated mammalian visual processing. This framework was used to train both single- and multi-resolution models to play the Atari game Pong. We combined a uniform low-resolution visual field and a small, cropped high-resolution visual field (fovea) as our multi-resolution visual input. The fovea was trained to track the ball using reinforcement learning.

Both single- and multi-resolution agents were trained with inputs with different characteristics, varying in resolution, history length, and foveal size. We statistically analyzed the agents' performance and resource usage under different scenarios. Our findings revealed that while the performance of both models is significantly impacted by the quality of the input, the multi-resolution model demonstrates a greater tolerance to lower-quality inputs and more efficient utilization of resources, such as neurons and synapses.

Our work contributes to a deeper understanding of the structural and functional efficiency of biological visual systems. It offers valuable insights into the design of visual perception networks for complex tasks and provides implications for applications of reinforcement learning, brain-like computing, and the development of adaptive AI systems. By bridging the gap between biological systems and artificial models, our study paves the way for advancements in both theoretical and practical aspects of sensorimotor learning.

## 2 Methods

### 2.1 The Pong environment

We chose Pong, one of the first computer games ever created, as the task for our reinforcement learning-based agent. Pong is a simple ping-pong-like game, featuring a ball and two paddles. One paddle is controlled by the game software, and the other is controlled by our agent. The goal for our agent is to play against the computer-controlled paddle and be the first one to gain 21 points (a player gets a point once the opponent misses a ball).

### 2.2 Peripheral and foveal visions

**Figure 2:**
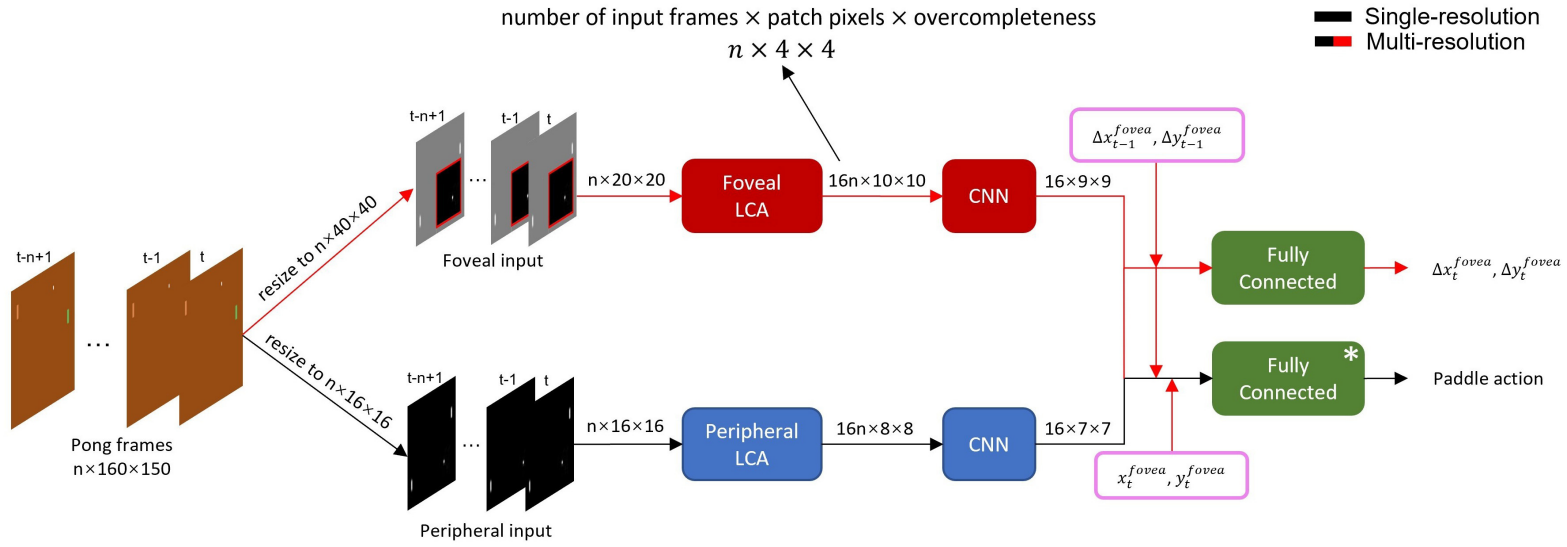
Detailed structure of single- and multi-resolution agents. Data flow of multi-resolution agent with 16 × 16 periphery, 20 × 20 fovea and *n*-frame input is shown as an example. The preprocessed frame sequence was input into corresponding foveal and peripheral LCA and CNN networks to extract spatiotemporal features. The single-resolution agent (black arrows) used only peripheral features (i.e. full frame at fixed resolution) to select paddle action. The multi-resolution agent (black and red arrows) combined peripheral and foveal features, fovea movement and position to select a paddle action and fovea movement in the next time step. *: The single-resolution agent had two fully connected layers to select paddle action while multi-resolution agent had three.

In this paper, we construct single- and multi-resolution agents, respectively, and compare their performance in the OpenAI Gym Pong environment (see Figure 1 for general information flow and Figure 2 for detailed structure). We examine high-acuity visual systems and pre-process Pong frames for two types of visual system: single-resolution peripheral (peripheral agent), and multi-resolution peripheral plus foveal vision (foveal agent). Our peripheral vision agent processed whole video frames, while our foveal vision agent received a part of the video frame at high resolution (40 × 40 pixels).

In our model, to mimic how animals use saccades to extend foveal vision, we made the fovea movable and thus it required a separate module to control its movements. Specifically, Pong frames were first converted to grayscale and then resized to a fixed resolution. For both peripheral and foveal agents, paddle and ball sizes were proportionally resized, while their relative positions were preserved. The peripheral agent's input was the entire preprocessed video frame. Then the *m* most recent frames were stacked giving time-dependent input to the agent. The foveal agent received cropped video frames according to the foveal position with the *n* most recent patches stacked giving local time-dependent input. Note that the stacked foveal patches may not have been cropped from the same physical area because the stacks included past foveal visual frames subject to time-dependent fovea movements.

### 2.3 Locally competitive algorithm

Visual inputs from both visual streams are first processed by the Locally Competitive Algorithm (LCA) (Rozell et al., 2008), a recurrent neural network for sparse coding and dictionary learning (see Figure 2). LCA uses recurrent inhibitory synaptic connectivity within a layer to competitively reduce activity in neuronal populations with small contributions to a reconstruction of its input. This results in a sparse representation of the input data. The dictionary of vectors capable of contributing to a sparse reconstruction is overcomplete, allowing flexibility to achieve sparse coding. Sparse coding via LCA is thought to be similar to how mammalian visual systems process visual information.

An LCA neuron with index *i* has internal state *u*_*i*_ and receives input, *a*_*i*_, through a synaptically encoded dictionary, *G*_*ij*_ described as


(1) $${\dot{u}}_{i}(t)=\frac{1}{\tau }[b_{i}-u_{i}(t)-\sum_{j\neq i}G_{ij}a_{j}(t)]$$

(2) $$a_{i}(t)=\{\begin{array}{ll} 0 & u_{i}(t)<\text{λ}\\ u_{i}(t) & u_{i}(t)\geq \text{λ} \end{array}$$

where *b*_*i*_ = 〈ϕ_*m*_, *s*〉 is an excitatory input current, and ϕ_*i*_ is the visual receptive field of neuron *i*. Lateral inhibition from neuron *j* to neuron *i* is proportional to *G*_*ij*_*a*_*j*_(*t*), where *G*_*ij*_ = 〈ϕ_*i*_, ϕ_*j*_〉. τ denotes a time constant and is set to 0.1. λ denotes threshold and is set to 1.25, 1.3, 1.35, 1.4 when the number of input frame is 1, 2, 3, 4, respectively. λ sets the degree of sparsity in the data reconstruction and here the effective sparsity(*N*_active neurons_/*N*_non-zero pixels_) is around 0.5 for 2, 3, 4 frame, and 0.8 for 1 frame.

**Figure 3:**
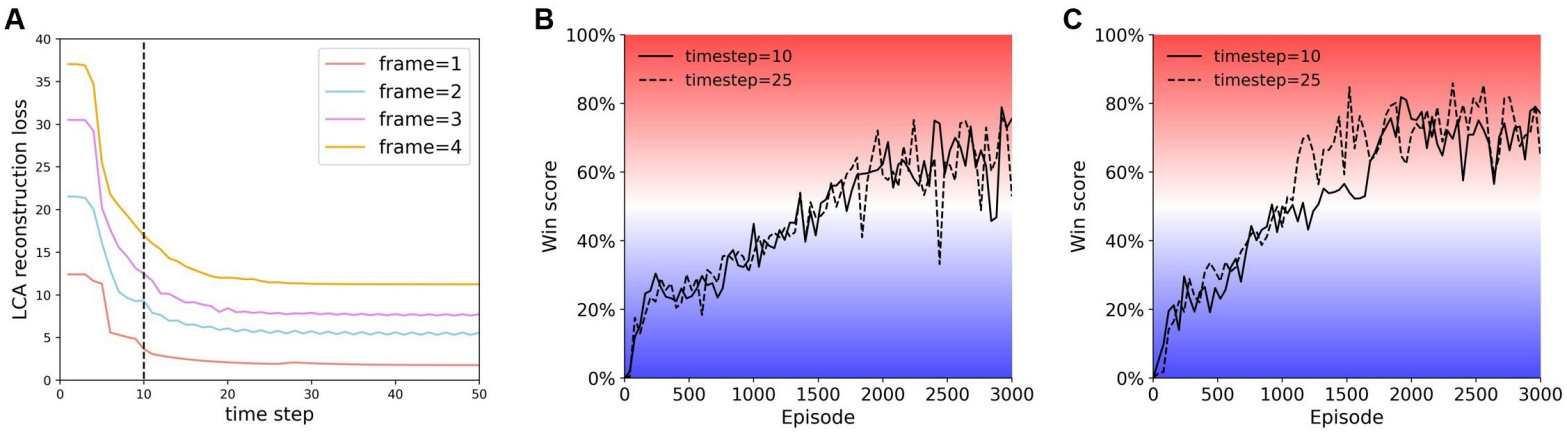
Comparing the LCA reconstruction loss at different times. **(A)** LCA reconstruction loss vs. time. Periphery = 40 × 40 for all reconstructions in this figure. **(B)** and **(C)** Training curves of agents trained at time step 10 and time step 25. **(B)** Single-resolution agent {40, 2}. **(C)** Multi-resolution agent {16, 12, 2}. Note that the agents train well at either time step 10 or 25.

The goal of the LCA algorithm is to approximately reconstruct the input, $\hat{s}(t)=\sum_{i}a_{i}(t)\phi_{i}\approx s(t)$. For each *n*-frame input, *u*_*i*_ and *a*_*i*_ will be updated according to Equations 1, 2 for 10 time steps (*dt* = 0.01). We show that the changes in LCA reconstruction loss as function of time have a similar tendency for different numbers of frames (Figure 3A). Though all curves in Figure 3A converge at 25 time steps, the difference in performance between 10 and 25 time steps is negligible (Figures 3B, C). Therefore, we set the number of time steps to 10 to reduce computation time. During training, receptive fields, ϕ_*i*_, are updated with the following rule,


(3) $$\begin{array}{l} \Delta \phi_{i}=\eta \left(s-ŝ\right)a_{i} \end{array}$$

where we used η = 0.002 as the learning rate.

In practice, visual inputs are divided into several non-overlapping patches of 2 × 2 pixels and processed by patch-based LCA, which shares weights between patches and encodes each patch independently. We use a 4 × overcomplete dictionary, such that, for *n*-frame input, there are 4 × 2 × 2 × *n* = 16*n* LCA neurons in each patch. We constrain dictionary elements to be unit norm. The peripheral LCA agent is trained first. LCA neurons for peripheral vision were trained with frames from Pong videos for 400 epochs, in which the agent paddle is controlled by random actions. In the first 200 epochs, we train half of the LCA neurons with frame patches from the vertical region that the paddle is constrained to move in. In the next 200 epochs, we fix these neurons and train the other half of the neurons with frame patches from all regions. This is done to capture information about the ball's motion. Subsequently, LCA neurons for foveal vision are trained on all neurons within fovea-sized images for 200 epochs, while concurrently training the foveal motion controller.

### 2.4 Deep-Q network

Both single- and multi-resolution agents (see Figures 1, 2) are based on deep Q-networks (DQN), which have been shown to be capable of playing many Atari games, at or better than human level, including Pong (Mnih et al., 2015). DQNs are based on a reinforcement learning paradigm that makes use of Q-learning (Watkins and Dayan, 1992). We use DQN to approximate the optimal action-value function


(4) $$\begin{array}{l} Q^{*}\left(s,a\right)=E_{s^{\prime }}\left[r+\gamma \underset{a^{\prime }}{\max}Q\left(s^{\prime },a^{\prime }\right)|s,a,\pi \right] \end{array}$$

where *r* is the reward, *s*′ and *a*′ are the state and action of the next time step, and π is the policy. γ is the discount factor which we set to be 0.99. At iteration *i*, we use a Q-learning update derived from the loss function


(5) $$\begin{array}{l} L_{i}\left(\theta_{i}\right)=E_{\left(s,a,r,s^{\prime }\right)\sim U\left(D\right)}\left[{\left(r+\gamma \underset{a^{\prime }}{\max}Q\left(s^{\prime },a^{\prime };\theta_{i}^{-}\right)-Q\left(s,a;\theta_{i}\right)\right)}^{2}\right] \end{array}$$

where θ_*i*_ are network parameters at iteration *i* and $\theta_{i}^{-}$ are the target network parameters at iteration *i*. The target network parameters, $\theta_{i}^{-}$, are only updated with DQN parameters, θ_*i*_, every 1, 000 steps and fixed between individual updates. *U*(*D*) denotes the experience pool which has a maximum size of 100, 000 samples for the foveal controller and 500, 000 for the action controller. During training, we use an epsilon greedy policy in which randomness of action selection begins with 100% and converges to 2%. The learning rate is set to be 0.0002. The foveal controller is trained for 300 epochs while the action controller is trained for 3, 000 epochs.

### 2.5 The foveal controller

To control foveal movement, we use reinforcement learning to train a foveal controller which outputs foveal movement at the next time step (Figure 2). In Pong, the position and velocity of the ball are the most critical information to win the game. Therefore, we design the foveal controller to track the ball and keep it in the center of the foveal vision. In the beginning of each game, the fovea will be fixed in the center of the Pong frame until the ball appears. The foveal controller is rewarded when the ball is in the center of the foveal vision field (4 × 4 pixels) and penalized when the ball is out of the foveal vision field. The reward function is summarized in Equation 6.


(6) $$r(t)=\{\begin{array}{ll} \;0.01 & \text{ if the ball is in the 2}\times \text{2 most central foveal patches}\\ \;-0.05 & \text{ if the ball is in the fovea},\text{but not in one of the 2}\times \text{2}\\ \; & \text{ most central patches}\\ \;-0.1 & \text{ if the ball is outside of fovea} \end{array}$$

The foveal controller has two branches, one for foveal movement along the *x*-axis ($\Delta x_{t}^{\text{fovea}}$) and the other for the *y*-axis ($\Delta y_{t}^{\text{fovea}}$). During training, the foveal controller produces two sets of *Q*-values. The loss value of each branch is computed individually according to Equation 5, *L*_*x*_(θ_*i*_) and *L*_*y*_(θ_*i*_). The training loss *L*_*i*_(θ_*i*_) is then their sum,


(7) $$\begin{array}{l} L_{i}\left(\theta_{i}\right)=L_{x}\left(\theta_{i}\right)+L_{y}\left(\theta_{i}\right). \end{array}$$

We use an agent wins about 50% of the time to play and record video frames when playing and use them as training data. The foveal controller combines visual information from both vision and foveal movement in the most recent time step to generate new foveal movement. The whole-frame resolution of foveal vision is fixed to be 40 × 40 pixels. When the foveal visual field runs off of the Pong screen, we augment the background. So, when the ball is close to the frame edge, the foveal controller can still keep the ball in its center and part of it can be outside of the Pong frame. The weights of the foveal controller, including CNN weights, are fixed when we train the action controller. Unlike other attention mechanisms (Xia et al., 2020; Chipka et al., 2021; Zhang et al., 2020; Killick et al., 2023; Lukanov et al., 2021; Liu et al., 2024), the foveal position in our model is continuous and updated based on foveal movement and its previous position.

### 2.6 The action controller

The ultimate objective for our agents is to be the first one to gain 21 points, so they need to be able to hit the ball back and not to miss it. We train models to map input states to *Q*-values, the expected reward, of each action. For the single-resolution agent (see Figure 2 black connections only), the only difference between it and a conventional DQN is that it has an LCA layer to pre-process peripheral inputs before convolutional layers. Peripheral LCA is trained and fixed before training other parts of single-resolution agents to identify the most valuable action of each state.

For the multi-resolution agent (see Figure 2 both black and red connections), the LCAs and the foveal controller are trained and fixed. When training to play Pong, the dense layers which output the paddle action receive not only visual information, but also the position coordinates, $x_{t}^{\text{fovea}}$ and $y_{t}^{\text{fovea}}$, and the velocity of the fovea, $\Delta x_{t-1}^{\text{fovea}}/\Delta t$ and $\Delta y_{t-1}^{\text{fovea}}/\Delta t$. Only the weights of the dense layers in the action controller are changed during this step of training for a multi-resolution agent.

## 3 Results

### 3.1 Tolerance to input quality in single- and multi-resolution agents

We examined how input quality affected the performance of single- and multi-resolution agents. We use win score *W* to evaluate agents, where


(8) $$\begin{array}{l} Win\;score=\frac{\text{Scor}{\text{e}}_{Agent}}{\text{Scor}{\text{e}}_{Agent}+\text{Scor}{\text{e}}_{Atari}}, \end{array}$$

where Score_*Agent*_ is the total number of points received by the RL agent, and Score_*Atari*_ is the total number of points that the OpenAI Gym Atari agent receives. For both agents, we varied the history length and input frame resolution. In addition, we studied different sizes of foveas in multi-resolution agents to test how foveal size affects performance.

**Figure 4:**
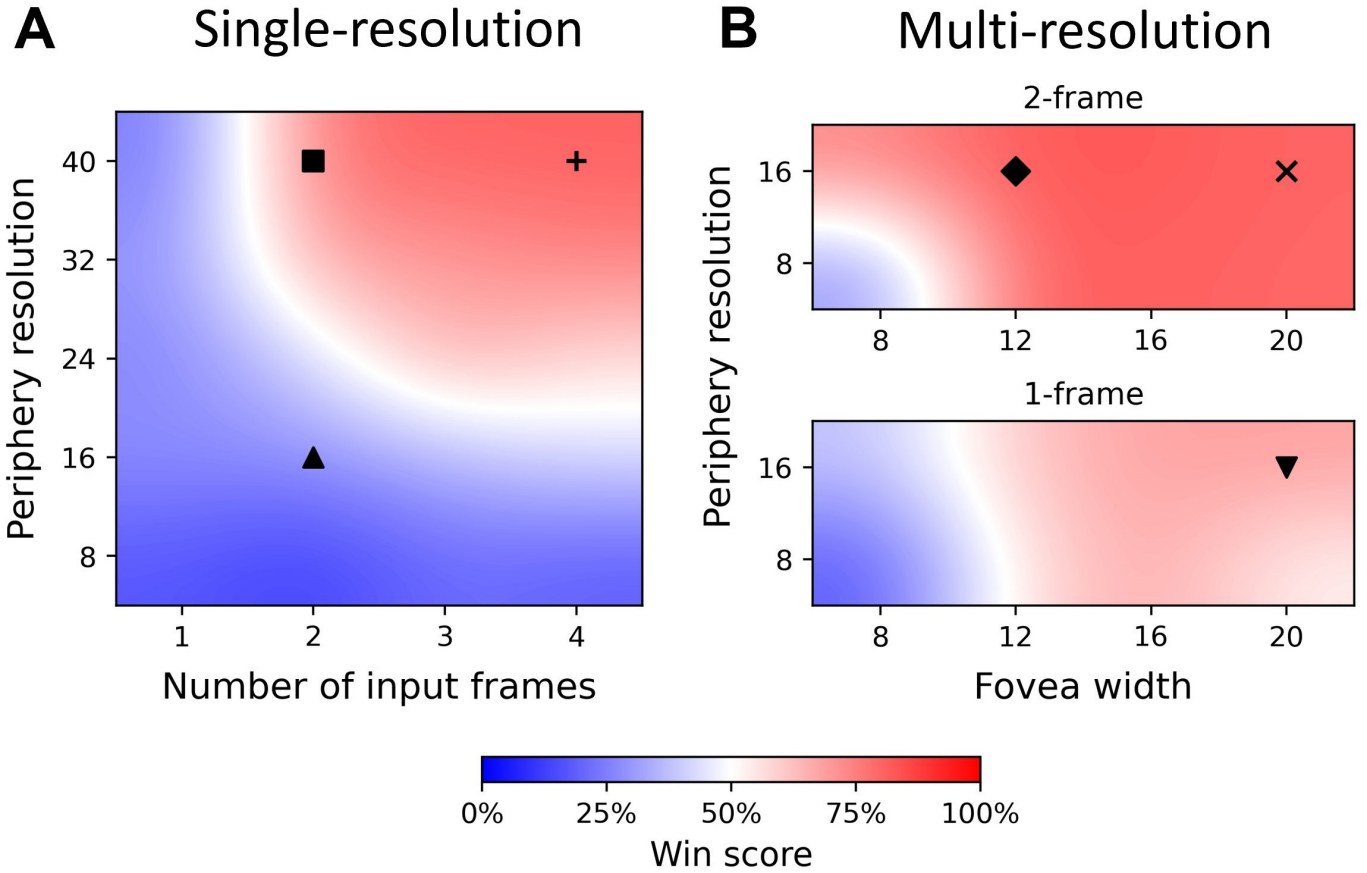
Win score heatmap of single- and multi-resolution agents showing differences in input quality. The best agent win scores are plotted and bicubic interpolation is used to fill other areas. The color bar denotes win score values. Markers (up triangle, square, plus, down triangle, diamond and *x*) in the heatmap correspond to agents with different input qualities and hyperparameters. Their training curves, test results and win score distributions are shown in Figures 5, 6. **(A)** Heatmap of single-resolution agent, exhibiting low win score with periphery ≤16 × 16 and 1-frame input, but higher win scores at high resolution and long history length. **(B)** Heatmap of multi-resolution model with 1 frame (bottom) and 2 frames (top) as input, exhibiting high win score even with periphery = 8 × 8 and 1-frame input.

**Figure 5:**
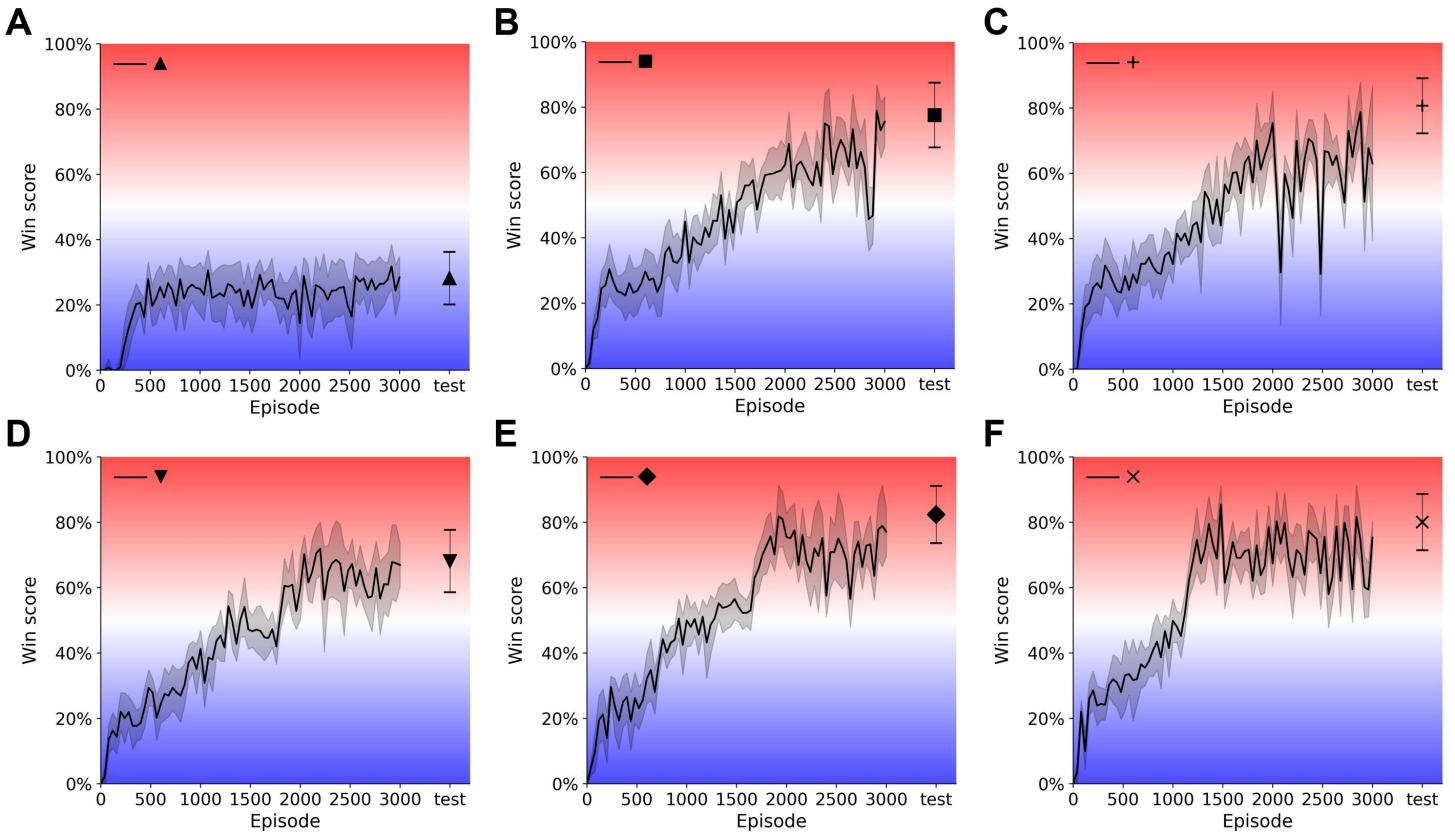
Training curves and test results of single- and multi-resolution agents. Model weights are saved every 40 episodes during training and evaluated for 20 games. Evaluation results are plotted with shading indicating standard deviation. Weights with the best evaluation result are tested for another 1,000 games and test results are plotted with error bar indicating standard deviation. Single-resolution agent {16, 2} **(A)**, {40, 2} **(B)**, {40, 4} **(C)**. Multi-resolution agent {16, 20, 1} **(D)**, {16, 12, 2} **(E)**, {16, 20, 2} **(F)**.

We trained and tested 36 agents with different sets of parameters. The results of these agents, which are the win score of the best agent with given settings, are shown in Figure 4. Training curves of 6 single- and multi-resolution agents are shown in Figure 5. Additionally, we assessed the best evaluation result by finding the maximum win score over 1, 000 subsequent test games (plotted to the right of the training curves in Figure 5). For simplification, we use {*r*_*p*_, *n*} to denote single-resolution agents with *r*_*p*_×*r*_*p*_ peripheral resolution and *n*-frame inputs, while we use {*r*_*p*_, *w*_*f*_, *n*} to denote multi-resolution agents with *r*_*p*_×*r*_*p*_ peripheral resolution, *w*_*f*_×*w*_*f*_ foveal size and *n*-frame inputs.

#### 3.1.1 Single-resolution agent

To compare single- and multi-resolution agents, we separated input quality into two dimensions: peripheral resolution and input frame number. We found that there is a clear boundary between regions of good (*W*>0.5) and bad (*W* < 0.5) performance (win score) (Figure 4A). When peripheral resolution is equal to or lower than 16 × 16, the single-resolution agent always performs poorly no matter how much we increase the history length. For instance, the performance of the single-resolution agent {16, 2} converges quickly and remains at a low level of around 20% throughout training (Figure 5A).

Even when we increased the history length to 4, the win score of the single-resolution agent {16, 4} is still below 50%. Similar behavior occurred when only the current frame was used as input. As the number of input frames and peripheral resolution increased across the boundary, the agent performed better and was able to achieve high win scores, which were around 80%. However, win scores could still fluctuate during training even when input quality was improved (longer history and/or higher resolution) (Figures 5B, C).

These results suggest that the performance of single-resolution agents is highly related to both dimensions of input quality. Defects in either dimension kept single-resolution agents in the low win score region, no matter how good the performance in the other dimension was. When peripheral resolution is too low, single-resolution agents cannot accurately determine the positions of the ball and paddles. Meanwhile, ball velocity was unpredictable for single-resolution agents when the input was only a single (i.e. the current) frame.

#### 3.1.2 Multi-resolution agent

Compared with single-resolution agents, multi-resolution agents showed a higher tolerance of low quality inputs and their performance compared better under similar input conditions. Since multi-resolution agents had foveas, we added foveal width as a new dimension of input quality. With their well-resolved foveas, multi-resolution agents achieved high win scores with only the current frame as input (Figure 4B, bottom). For example, after training for about 2000 episodes, the win score of the multi-resolution agent {16, 20, 1} reached 70% (Figure 5D), which was about 25% higher than the single-resolution agent {16, 4} with the same peripheral resolution. This was also 25% higher than the single-resolution agent {24, 2}, which used a similar number of pixels.

We attributed the lower input quality tolerance of multi-resolution agents to the fovea. Multi-resolution agents used their small, but high resolution, fovea to track the ball such that it could capture both the position and velocity of the ball accurately with low peripheral resolution. Additional inputs, such as foveal movement and position, had a similar effect to memory, helping the agent to counteract the lack of temporal information of a single input frame. Moreover, pre-trained CNNs were able to capture and combine both global and local features from both components of the visual system to make a better prediction of the expected reward of each action.

As input quality improved, multi-resolution agents still benefited from their design. With 2-frame input, multi-resolution agents always had excellent performance for most sets of hyperparameters (Figure 4B, top) while single-resolution agents only performed well when peripheral resolution was equal to or higher than 32 × 32. Multi-resolution agents showed higher pixel use under these circumstances. For instance, comparing with the single resolution agent {40, 2}, the multi-resolution agent {16, 12, 2} used only 25% of its pixels to achieve a 5% higher win score. This multi-resolution agent converged over 200 episodes earlier than the single-resolution agent with smaller fluctuations during training (Figure 5E). Similar things happened when the input quality improved and multi-resolution agents converged even faster, training in about 1, 200 episodes (Figure 5F). Though multi-resolution agents were efficient in using input information, poor quality input still influenced their performance. Except for {16, 8, 2}, other agents with 8 × 8 foveas could not achieve good performance. Meanwhile, multi-resolution agents with 16 × 16 peripheral resolution always performed better than those with 8 × 8 peripheral resolution when other conditions were the same.

#### 3.1.3 Distribution of the win score

**Figure 6:**
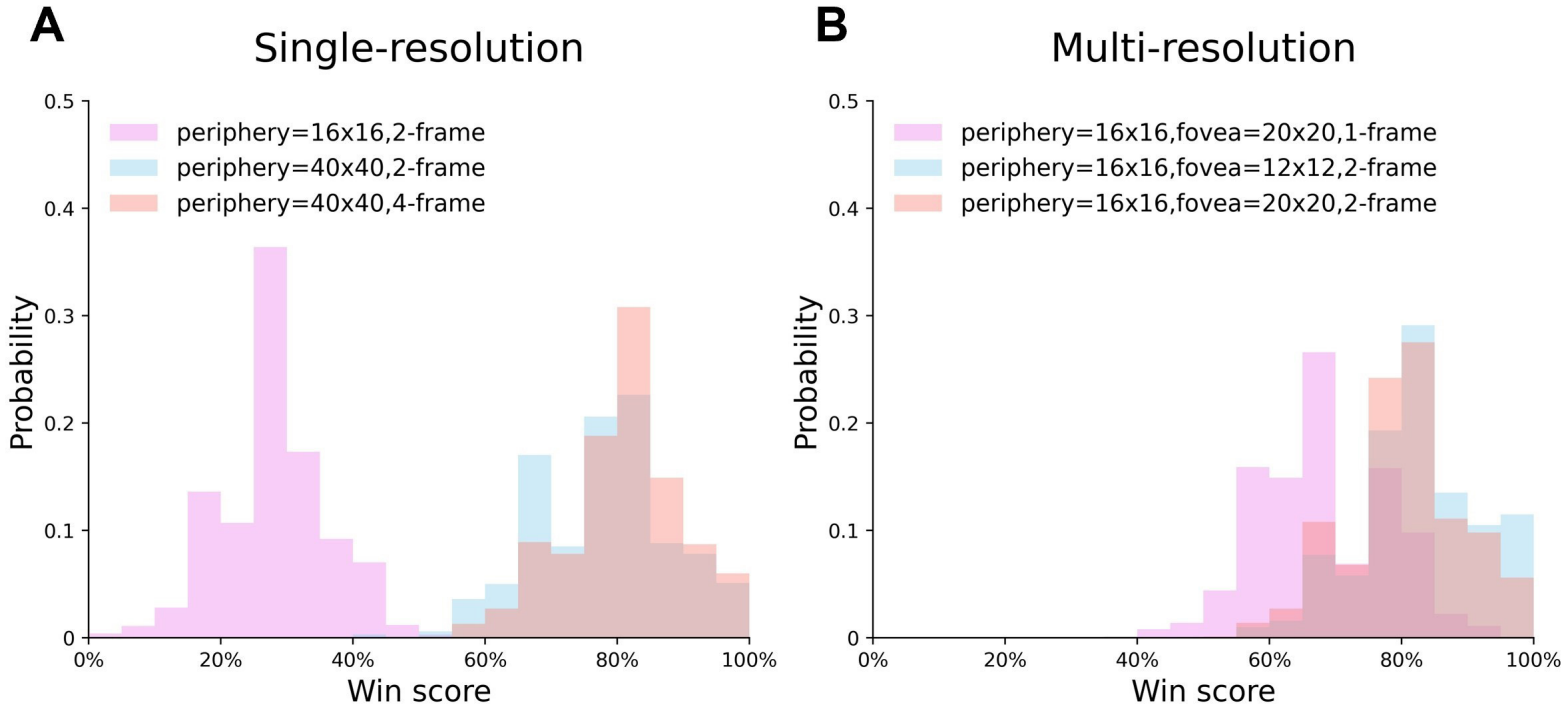
The distribution of single- and multi-resolution agents' test results. **(A)** Single-resolution agent. **(B)** Multi-resolution agent.

In Figure 6, we investigate the win score distribution of six best-performing single- and multi-resolution agents, whose training curves are shown in Figure 5. The result shows that the single-resolution agent {16, 2} almost never obtained a win score above 50% (Figure 6A, violet), which means it always lost. However, the multi-resolution agent with 1-frame input won the most test games (Figure 6B, violet). This result suggested that compared to single-resolution agents, multi-resolution agents can extract more information from low quality input and have higher tolerance to low quality input. As the input quality improved, the win score distributions of both single- and multi-resolution agents shifted to the right and always had a win score that was higher than 50%, indicating that both agents could benefit from the better input quality and have good stability when the mean win score was high.

### 3.2 Resource utilization in single- and multi-resolution agents

To explore the resource efficiency of both kinds of agents, we compare the resource use and win score of agents as a function of different hyperparameters. More specifically, the number of neurons and synapses in the network, and a measure of computational throughput (Floating Point Operations Per Second, FLOPS) are used for comparison. Notice that, for our multi-resolution agent, foveal movement and paddle action were generated by the same visual information. Therefore, the generation of foveal movement does not need extra resources to process visual information.

When peripheral resolution is low or the number of input frames is small, single-resolution agents perform poorly (Table 1 {16, 4} and {40, 1}). However, with input that has a longer history but lower resolution, these agents can achieve significantly higher win scores using fewer neurons and synapses (compare Table 1 {40, 1} with {24, 3}). This suggests that for the same network architecture, investing more resources might not lead to better performance if the resources are used in the wrong place. Improving input quality in the correct dimension (*i.e*. spatial scale vs. history length) can make the model perform significantly better, even with fewer resources.

**Table 1:** Win scores of single-resolution agent using inputs of different quality and resources.

**Periphery resolution**	**Input frames**	**Neurons**	**Synapses**	**FLOPs**	**Win score**
40	1	9.51 × 10^3^	2.10 × 10^6^	6.21 × 10^6^	24.0%
16	4	4.90 × 10^3^	7.08 × 10^5^	6.34 × 10^6^	43.2%
24	3	8.23 × 10^3^	1.36 × 10^6^	8.90 × 10^6^	67.2%
40	4	2.87 × 10^4^	5.62 × 10^6^	4.20 × 10^7^	80.7%

With only the current frame as input, the win score of multi-resolution agents is 18% higher than single-resolution agents that use similar numbers of synapses but more neurons and computational throughput (compare Table 2 {16, 12, 1} with Table 1 {16, 4}). Increasing the number of input frames leads to better performance than increasing peripheral resolution (compare Table 2 {8, 12, 1}, {16, 12, 2} and {8, 12, 2}). This agrees with the results seen with single-resolution agents. Furthermore, multi-resolution agents need fewer resources to stabilize their training curves when both single- and multi-resolution agents can achieve comparable performance (compare Table 2 {16, 12, 2} and Table 1 {40, 4}).

**Table 2:** Win scores of Multi-resolution agent using inputs of different quality and resources.

**Periphery resolution**	**Fovea width**	**Input frames**	**Neurons**	**Synapses**	**FLOPs**	**Win score**
8	12	1	2.03 × 10^3^	4.00 × 10^5^	1.06 × 10^6^	55.4%
16	12	1	3.44 × 10^3^	7.86 × 10^5^	2.08 × 10^6^	61.4%
8	12	2	2.86 × 10^3^	4.74 × 10^5^	1.97 × 10^6^	80.2%
16	12	2	6.32 × 10^3^	9.37 × 10^5^	3.84 × 10^6^	82.4%

**Figure 7:**
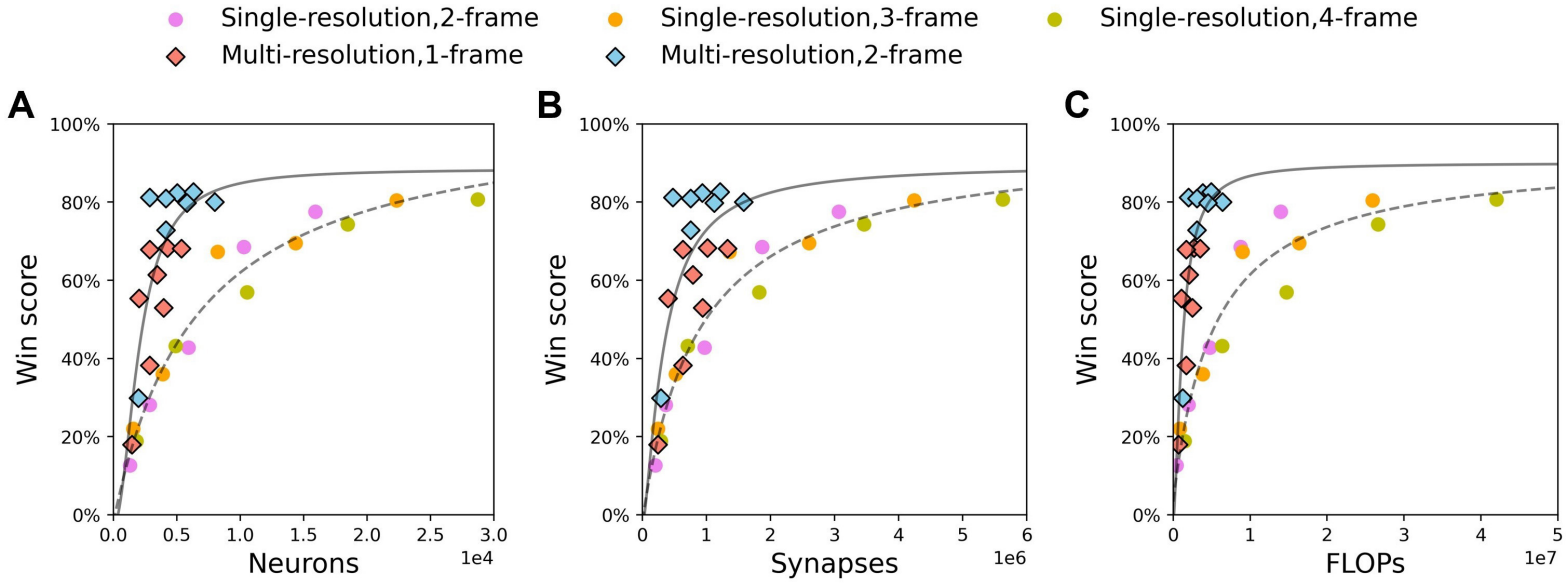
Multi-resolution agents using fewer resources than single-resolution agent to achieve the same win score. Circle: single-resolution agent. Diamond: multi-resolution agent. Function $y=a-\frac{b}{x^{c}+d}$ is used to fit win scores of single-resolution agent (dash line) and multi-resolution agent (solid line). **(A)** Neurons. **(B)** Synapses. **(C)** FLOPs.

We show results of single-resolution agents with 2, 3, and 4-frame input and multi-resolution agents with 1 and 2-frame input in Figure 7. For each resource, win scores for each agent are fitted with the functional form $y=a-\frac{b}{x^{c}+d}$, where *y* denotes win score, *x* denotes the resource and (*a, b, c, d*) are fit parameters. It is clear that for all resources, the fitting curve of multi-resolution agents is above that of single-resolution agents for all resource amounts. This indicates that multi-resolution agents have higher efficiency in resource utilization. Combining with results in Tables 1, 2, this indicates that low resolution could be one of the reasons for high efficiency. It is interesting that the win scores of single-resolution agents roughly follow the same curves as resources change, even though the history length for these agents is different. However, the results of multi-resolution agents show that when using similar amounts of resources, agents with longer history lengths always perform better than agents with shorter histories (Figure 7, red and blue diamonds). This implies that comparing with single-resolution agents, multi-resolution agents have a better ability to extract information from input with long history lengths. This could be one of the reasons that multi-resolution agents use resources more efficiently.

## 4 Discussion and conclusions

In this paper, we trained biologically-motivated agents, with and without a fovea, to play Pong via reinforcement learning. We then compared their performance and resource utilization. We showed that the performance of agents with a fovea converges faster during training and is more stable after the agent achieves a high win score. At the same time, the agent with a fovea has a higher tolerance to lower input quality and uses resources more efficiently. This includes the number of neurons, synapses, and computations.

Comparing with other reinforcement learning agents that did not use LCA, we found that our multi-resolution agent had a higher pixel-efficiency. For example, the DQN RL agent in Mnih et al. (2015) achieved a 91% win score in Pong, which was about 8% higher than the mean score of our multi-resolution agent {16, 12, 2} and about equal to its best score (see error bars on the right of Figure 5D). However, our agent used about 3% of the pixels used in Mnih et al. (2015). Similarly, in Adhikari and Ren (2021), the authors used a Double DQN agent achieving similar win scores with our multi-resolution agent {16, 12, 2}, while our agent only used 2% of the pixels used in the Double DQN agent.

To emulate visual processing in mammalian primary visual cortex (V1), we used LCA to preprocess the visual inputs from peripheral and foveal vision. In studies of V1, researchers have discovered that the spatiotemporal characteristics of V1 receptive fields can be represented by a dictionary tuned to give rise, via recurrent connections, to sparse responses to natural images (Olshausen, 2003). Recordings of V1 activity under natural scene stimulation have revealed that neural activity becomes sparse when adjacent neurons are stimulated (Vinje and Gallant, 2002). Additionally, it has been found experimentally that the dictionary of V1 receptive fields is overcomplete (Olshausen and Field, 2004), meaning that the number of neurons in V1 exceeds the dimensionality of the input signals. This overcompleteness confers a high degree of flexibility in representing input information. Such flexible sparse coding confers numerous advantages to the sensory nervous system, including enhanced performance in subsequent processing stages, increased memory capacity, and improved energy efficiency. The locally competitive algorithm contains all these properties, including lateral inhibition and overcompleteness, which make it a candidate for sparse coding in the visual system.

Many studies transform LCA into a spiking neural network and use it as a benchmark to run on neuromorphic hardware (Woods et al., 2015; Fair et al., 2019; Parpart et al., 2023; Hong et al., 2024). Neuromorphic systems are event-based and they consume energy only when spikes are generated. Compared to traditional computing systems, neuromorphic systems require only about 1% of the energy to run the same algorithm (Göltz et al., 2021), which makes it perfect for devices with low SWaP (size, weight, and power) requirements. Moreover, a neuromorphic LCA algorithm capable of learning a dictionary has been demonstrated (Chavez Arana et al., 2023). Hence, this early visual part of our algorithm is capable of a low SWaP implementation.

The later visual system is represented by deep nets (i.e. feedforward neural networks with many layers). A recent study proposes a neuromorphic backpropagation algorithm implemented on spiking neuromorphic hardware (Renner et al., 2024) that uses mechanisms that have been experimentally demonstrated in mammalian cortex, such as synfire chains (Mao et al., 2001; Ikegaya et al., 2004) and communication through coherence (Riehle et al., 1997; Sornborger et al., 2015; Wang et al., 2016). Thus, the deep net part of our vision learning framework can also be implemented neuromorphically. Taken together, this implies that a learning, spiking implementation of our framework for learning Pong is feasible.

The non-uniform spatial distribution with which we sampled visual information has analogs in other sensory modalities. For example, in human skin, the distribution of mechanoreceptors is not uniform (Johnson, 2001). Mechanoreceptors are denser in the fingertips, which are the main body parts that we actively use to touch objects. In the human auditory system, the range of human hearing is from 20 to 20, 000 Hz. But due to the distribution of inner hair cells in the cochlea, a human is more sensitive to frequencies between 2, 000 and 5, 000 Hz (Gelfand, 2009). This range contains the bulk of the frequencies relevant to human speech. Different sensory systems focus most of their resources on perceiving key local information while maintaining a global awareness, which helps organisms acquire information more efficiently.

To play Pong, our agents combined information from different parts of the visual system, the fovea and the periphery. One possible improvement for our agent might be to add other sensory modalities, such as sound. Adding a new sensory modality may supply complementary information and provide a more comprehensive view of a task. For example, in order to immerse the player in the game, background music in video games always provides supplementary information about the current game state and even foreshadows what is going to happen. From the point of view of neuroscience, multisensory integration, or the binding problem, has gained a lot of attention (Roskies, 1999; Zmigrod and Hommel, 2010; Powers III et al., 2016; Yu and Lau, 2023). Studying how a biologically-plausible multisensory agent solves complex decision-making tasks, like games, is a possible way to study how binding happens in biological neural networks.

## Data Availability

The raw data supporting the conclusions of this article will be made available by the authors, without undue reservation.

## References

[B1] Adhikari A. Ren Y. (2021). RL-PONG: Playing Pong From Pixels. Project Proposal, CSCE 790-001: Deep Reinforcement Learning and Search.

[B2] Baker B. Akkaya I. Zhokov P. Huizinga J. Tang J. Ecoffet A.. (2022). Video pretraining (VPT): learning to act by watching unlabeled online videos. Adv. Neural Inf. Process. Syst. 35, 24639–24654. 10.48550/arXiv.2206.11795

[B3] Bringmann A. (2019). Structure and function of the bird fovea. Anat. Histol. Embryol. 48, 177–200. 10.1111/ahe.12432 30734347

[B4] Bringmann A. Syrbe S. Görner K. Kacza J. Francke M. Wiedemann P. Reichenbach A. (2018). The primate fovea: structure, function and development. Prog. Retin. Eye Res. 66, 49–84. 10.1016/j.preteyeres.2018.03.006 29609042

[B5] Chavez Arana D. Renner A. Sornborger A. (2023). “Spiking LCA in a neural circuit with dictionary learning and synaptic normalization,” in Proceedings of the 2023 Annual Neuro-Inspired Computational Elements Conference (New York, NY: ACM), 47–51. 10.1145/3584954.3584968

[B6] Chipka J. Zeng S. Elvitigala T. Mudalige P. (2021). “A computer vision-based attention generator using DQN,” in Proceedings of the IEEE/CVF International Conference on Computer Vision (Montreal, BC IEEE), 2942–2950. 10.1109/ICCVW54120.2021.00329

[B7] Day J. J. Roitman M. F. Wightman R. M. Carelli R. M. (2007). Associative learning mediates dynamic shifts in dopamine signaling in the nucleus accumbens. Nat. Neurosci. 10, 1020–1028. 10.1038/nn1923 17603481

[B8] Fair K. L. Mendat D. R. Andreou A. G. Rozell C. J. Romberg J. Anderson D. V.. (2019). Sparse coding using the locally competitive algorithm on the TrueNorth neurosynaptic system. Front. Neurosci. 13:754. 10.3389/fnins.2019.00754 31396039 PMC6664083

[B9] Gelfand S. (2009). Essentials of Audiology. Thieme Publishers Series. New York, NY: Thieme.

[B10] Göltz J. Kriener L. Baumbach A. Billaudelle S. Breitwieser O. Cramer B.. (2021). Fast and energy-efficient neuromorphic deep learning with first-spike times. Nat. Mach. Intell. 3, 823–835. 10.1038/s42256-021-00388-x37523463

[B11] Holtmaat A. Svoboda K. (2009). Experience-dependent structural synaptic plasticity in the mammalian brain. Nat. Rev. Neurosci. 10, 647–658. 10.1038/nrn2699 19693029

[B12] Hong Q. Xiao P. Fan R. Du S. (2024). Memristive neural network circuit design based on locally competitive algorithm for sparse coding application. Neurocomputing 578:127369. 10.1016/j.neucom.2024.127369

[B13] Hübener M. Bonhoeffer T. (2014). Neuronal plasticity: beyond the critical period. Cell 159, 727–737. 10.1016/j.cell.2014.10.035 25417151

[B14] Ikegaya Y. Aaron G. Cossart R. Aronov D. Lampl I. Ferster D.. (2004). Synfire chains and cortical songs: temporal modules of cortical activity. Science 304, 559–564. 10.1126/science.1093173 15105494

[B15] Johnson K. O. (2001). The roles and functions of cutaneous mechanoreceptors. Curr. Opin. Neurobiol. 11, 455–461. 10.1016/S0959-4388(00)00234-8 11502392

[B16] Kable J. W. Glimcher P. W. (2009). The neurobiology of decision: consensus and controversy. Neuron 63, 733–745. 10.1016/j.neuron.2009.09.003 19778504 PMC2765926

[B17] Killick G. Henderson P. Siebert P. Aragon-Camarasa G. (2023). Foveation in the era of deep learning. arXiv [Preprint]. arXiv:2312.01450. 10.48550/arXiv.2312.01450

[B18] Knutson B. Delgado M. R. Phillips P. E. (2009). “Representation of subjective value in the striatum,” in Neuroeconomics, eds. P. W. Glimcher, C. F. Camerer, E. Fehr, and Russell A. Poldrack (Amsterdam: Elsevier), 389–406. 10.1016/B978-0-12-374176-9.00025-7

[B19] Land M. Eckert H. (1985). Maps of the acute zones of fly eyes. J. Comp. Physiol. A 156, 525–538. 10.1007/BF00613976

[B20] Lee D. Seo H. Jung M. W. (2012). Neural basis of reinforcement learning and decision making. Annu. Rev. Neurosci. 35, 287–308. 10.1146/annurev-neuro-062111-150512 22462543 PMC3490621

[B21] Liu J. Bu Y. Tso D. Qiu Q. (2024). “Improved efficiency based on learned saccade and continuous scene reconstruction from foveated visual sampling,” in Twelfth International Conference on Learning Representations.

[B22] Lukanov H. König P. Pipa G. (2021). Biologically inspired deep learning model for efficient foveal-peripheral vision. Front. Comput. Neurosci. 15:746204. 10.3389/fncom.2021.746204 34880741 PMC8645638

[B23] Mao B-. Q. Hamzei-Sichani F. Aronov D. Froemke R. C. Yuste R. (2001). Dynamics of spontaneous activity in neocortical slices. Neuron 32, 883–898. 10.1016/S0896-6273(01)00518-9 11738033

[B24] Min J. Zhao Y. Luo C. Cho M. (2022). Peripheral vision transformer. Adv. Neural Inf. Process. Syst. 35, 32097–32111.

[B25] Mirhoseini A. Goldie A. Yazgan M. Jiang J. W. Songhori E. Wang S.. (2021). A graph placement methodology for fast chip design. Nature 594, 207–212. 10.1038/s41586-021-03544-w 34108699

[B26] Mnih V. (2013). Playing Atari with deep reinforcement learning. arXiv [Preprint]. arXiv:1312.5602. 10.48550/arXiv.1312.5602

[B27] Mnih V. Kavukcuoglu K. Silver D. Rusu A. A. Veness J. Bellemare M. G.. (2015). Human-level control through deep reinforcement learning. Nature 518, 529–533. 10.1038/nature14236 25719670

[B28] Montague P. Dayan P. Nowlan S. J. Pouget A. Sejnowski T. (1992). Using aperiodic reinforcement for directed self-organization during development. Adv. Neural Inf. Process. Syst. 5, 969–976.

[B29] Montague P. R. Dayan P. Sejnowski T. J. (1996). A framework for mesencephalic dopamine systems based on predictive hebbian learning. J. Neurosci. 16, 1936–1947. 10.1523/JNEUROSCI.16-05-01936.1996 8774460 PMC6578666

[B30] Nguyen H. La H. (2019). “Review of deep reinforcement learning for robot manipulation,” in 2019 Third IEEE International Conference on Robotic Computing (IRC) (Naples: IEEE), 590–595. 10.1109/IRC.2019.00120

[B31] Niv Y. (2009). Reinforcement learning in the brain. J. Math. Psychol. 53, 139–154. 10.1016/j.jmp.2008.12.005

[B32] Olshausen B. A. (2003). Principles of image representation in visual cortex. Vis. Neurosci. 2, 1603–1615. 10.7551/mitpress/7131.003.0123

[B33] Olshausen B. A. Field D. J. (2004). Sparse coding of sensory inputs. Curr. Opin. Neurobiol. 14, 481–487. 10.1016/j.conb.2004.07.007 15321069

[B34] Parpart G. Risbud S. Kenyon G. Watkins Y. (2023). “Implementing and benchmarking the locally competitive algorithm on the Loihi 2 neuromorphic processor,” in Proceedings of the 2023 International Conference on Neuromorphic Systems (New York, NY: IEEE), 1–6. 10.1145/3589737.3605973

[B35] Powers I. I. I. Hillock-Dunn A. R. Wallace A. M. T. (2016). Generalization of multisensory perceptual learning. Sci. Rep. 6:23374. 10.1038/srep23374 27000988 PMC4802214

[B36] Renner A. Sheldon F. Zlotnik A. Tao L. Sornborger A. (2024). The backpropagation algorithm implemented on spiking neuromorphic hardware. Nat. Commun. 15:9691. 10.1038/s41467-024-53827-9 39516210 PMC11549378

[B37] Riehle A. Grun S. Diesmann M. Aertsen A. (1997). Spike synchronization and rate modulation differentially involved in motor cortical function. Science 278, 1950–1953. 10.1126/science.278.5345.1950 9395398

[B38] Rong Y. Xu W. Akata Z. Kasneci E. (2021). Human attention in fine-grained classification. arXiv [Preprint]. arXiv:2111.01628. 10.48550/arXiv.2111.01628

[B39] Roskies A. L. (1999). The binding problem. Neuron 24, 7–9. 10.1016/S0896-6273(00)80817-X 10677022

[B40] Rozell C. J. Johnson D. H. Baraniuk R. G. Olshausen B. A. (2008). Sparse coding via thresholding and local competition in neural circuits. Neural Comput. 20, 2526–2563. 10.1162/neco.2008.03-07-486 18439138

[B41] Schultz W. Dayan P. Montague P. R. (1997). A neural substrate of prediction and reward. Science 275, 1593–1599. 10.1126/science.275.5306.1593 9054347

[B42] Sornborger A. T. Wang Z. Tao L. (2015). A mechanism for graded, dynamically routable current propagation in pulse-gated synfire chains and implications for information coding. J. Comput. Neurosci. 39, 181–195. 10.1007/s10827-015-0570-8 26227067 PMC4659484

[B43] Thakur R. K. Sunbeam M. Goecks V. G. Novoseller E. Bera R. Lawhern V. J.. (2021). Imitation learning with human eye gaze via multi-objective prediction. arXiv [Preprint]. arXiv:2102.13008. 10.48550/arXiv.2102.13008

[B44] Traver V. J. Bernardino A. (2010). A review of log-polar imaging for visual perception in robotics. Rob. Auton. Syst. 58, 378–398. 10.1016/j.robot.2009.10.002

[B45] Vinje W. E. Gallant J. L. (2002). Natural stimulation of the nonclassical receptive field increases information transmission efficiency in V1. J. Neurosci. 22, 2904–2915. 10.1523/JNEUROSCI.22-07-02904.2002 11923455 PMC6758304

[B46] Vinyals O. Babuschkin I. Czarnecki W. M. Mathieu M. Dudzik A. Chung J.. (2019). Grandmaster level in starcraft ii using multi-agent reinforcement learning. Nature 575, 350–354. 10.1038/s41586-019-1724-z 31666705

[B47] Wang Z. Sornborger A. T. Tao L. (2016). Graded, dynamically routable information processing with synfire-gated synfire chains. PLoS Comput. Biol. 12:e1004979. 10.1371/journal.pcbi.1004979 27310184 PMC4911121

[B48] Watkins C. J. Dayan P. (1992). Q-learning. Mach. Learn. 8, 279–292. 10.1007/BF00992698

[B49] Wiesel T. N. Hubel D. H. (1963). Single-cell responses in striate cortex of kittens deprived of vision in one eye. J. Neurophysiol. 26, 1003–1017. 10.1152/jn.1963.26.6.1003 14084161

[B50] Woods W. Bürger J. Teuscher C. (2015). Synaptic weight states in a locally competitive algorithm for neuromorphic memristive hardware. IEEE Trans. Nanotechnol. 14, 945–953. 10.1109/TNANO.2015.2449835

[B51] Wurman P. R. Barrett S. Kawamoto K. MacGlashan J. Subramanian K. Walsh T. J.. (2022). Outracing champion Gran Turismo drivers with deep reinforcement learning. Nature 602, 223–228. 10.1038/s41586-021-04357-7 35140384

[B52] Xia Y. Kim J. Canny J. Zipser K. Canas-Bajo T. Whitney D.. (2020). “Periphery-fovea multi-resolution driving model guided by human attention,” in Proceedings of the IEEE/CVF Winter Conference on Applications of Computer Vision (Snowmass, CO: IEEE), 1767–1775. 10.1109/WACV45572.2020.9093524

[B53] Yu X. Lau E. (2023). The binding problem 2.0: beyond perceptual features. Cogn. Sci. 47:e13244. 10.1111/cogs.13244 36744750

[B54] Zhang R. Walshe C. Liu Z. Guan L. Muller K. Whritner J.. (2020). Atari-head: Atari human eye-tracking and demonstration dataset. Proc. AAAI Conf. Artif. Intell. 34, 6811–6820. 10.1609/aaai.v34i04.6161 32901213 PMC7476327

[B55] Zmigrod S. Hommel B. (2010). Temporal dynamics of unimodal and multimodal feature binding. Attent. Percept. Psychophys. 72, 142–152. 10.3758/APP.72.1.142 20045885

