# Supplementary material for: Foveal vision reduces neural resources in agent-based game learning
Source: Front Neurosci. 2025 Mar 11;19:1547264. doi: 10.3389/fnins.2025.1547264 (PMC11933080; doi:10.3389/fnins.2025.1547264)
Supplement: Supplementary file 1 [file Data_Sheet_1.pdf]

# Supplementary Material

## 1 CALCULATION OF RESOURCES

In this section we outline the methods we used to calculate agent resources, including the numbers of neurons, numbers of synapses and FLOPs. Most of our calculations were done by hand. This was because the definition of neurons and synapses comes from neuroscience, so using nodes and parameters calculated with a Python function could be incorrect. Additionally, LCA is not a conventional artificial neural network so there is no Python function to calculate its resources.

In the following calculation, we let input image resolution $= r \times r$, overcompleteness $= o$, number of input frames $= n_f$, number of CNN kernels $= n_k$, kernel size $= s_k \times s_k$, kernel stride $= d_k$, and LCA patch size $= s_p \times s_p$.

### 1.1 Neuron

By definition, the number of LCA neurons $N_{\text{LCA}}$ is the number of input pixels times overcompleteness:

$$N_{\text{LCA}} = n_f r^2 \cdot o \tag{S1}$$

The number of CNN neurons $N_{\text{CNN}}$ is calculated by

$$N_{\text{CNN}} = n_k \left( \frac{\frac{r}{s_p} - s_k + d_k}{d_k} \right)^2 \tag{S2}$$

The number of all-to-all neurons $N_{\text{all}}$ is equal to the number of nodes in all-to-all layers.

### 1.2 Synapse

The number of synapses inside the LCA network $S_{\text{LCA\_inside}}$ is the number of synapses in each LCA patch times the number of LCA patches:

$$S_{\text{LCA\_inside}} = n_f\, s_p^2\, o \left( n_f\, s_p^2\, o - 1 \right) \cdot \left( \frac{r}{s_p} \right)^2 \tag{S3}$$

Notice that LCA neurons do not have self-connections.
The number of LCA-CNN synapses $S_{\text{LCA}-\text{CNN}}$ is equal to the number of neurons in each LCA patch times kernel size times the number of neurons in CNN:

$$S_{\text{LCA}-\text{CNN}} = n_f\, s_p^2\, o \cdot s_k^2 \cdot N_{\text{CNN}} \tag{S4}$$

where the number of neurons in each LCA patch is used as the number of channels of the CNN. The number of CNN-all and all-all synapses is easy to calculate because they are all all-to-all connections.

## 1.3 FLOPs

We calculated the computations performed by the LCA network with the definition of a FLOP. A floating-point operation (FLOP) is any mathematical operation (such as +, -, *, /) or assignment that involves floating-point numbers. First, to get excitatory input current, $b$, and inhibition weights, $G$, we needed to perform two matrix multiplications, which were input times the dictionary matrix and the dictionary matrix times transposed dictionary. So the FLOPs in this stage were

$$F_{LCA1} = 2 \cdot [\left(\frac{r}{s_p}\right)^2 \cdot n_f\ s_p^2 \cdot n_f\ s_p^2\ o + n_f\ s_p^2\ o \cdot n_f\ s_p^2 \cdot n_f\ s_p^2\ o] = 2n_f^2\ r^2\ s_p^2\ o + 2n_f^3\ s_p^3\ o^2 \quad \text{(S5)}$$

Then, the next stage is to update LCA potential $u$ for ten timesteps according to

$$\dot{u}_i(t) = \frac{1}{\tau}[b_i - u_i(t) - \sum_{j \neq i} G_{ij} a_j(t)] \quad \text{(S6)}$$

So the FLOPs at this stage may be evaluated with

$$F_{LCA2} = 10 \cdot (3N_{LCA} + 2 \cdot N_{LCA} \cdot n_f s_p^2 o) \quad \text{(S7)}$$

The FLOPs of the CNN and the fully connected layers can be calculate with the function *profile* in Python library *thop*. Notice that the unit of computation output of *profile* is MAC, which signifies a Multiply-Accumulate Operation. 1 MAC is equal to 2 FLOPs.
